# Hierarchical Natural Fibre Composites Based on Cellulose Nanocrystal-Modified Luffa Structures for Binderless Acoustic Panels

**DOI:** 10.3390/polym17030281

**Published:** 2025-01-22

**Authors:** Shahed Ekbatani, Phattharasaya Rattanawongkun, Supattra Klayya, Dimitrios G. Papageorgiou, Nattakan Soykeabkaew, Han Zhang

**Affiliations:** 1 School of Engineering and Materials Science, Queen Mary University of London, London E1 4NS, UK; s.ekbatani@qmul.ac.uk (S.E.); d.papageorgiou@qmul.ac.uk (D.G.P.); 2 Center of Innovative Materials for Sustainability (iMatS), School of Science, Mae Fah Luang University, 333 M1, Muang, Chiang Rai 57100, Thailand; 6771108001@lamduan.mfu.ac.th (P.R.); 6571108001@lamduan.mfu.ac.th (S.K.); nattakan@mfu.ac.th (N.S.)

**Keywords:** luffa fibres, porous structure, sound absorption, acoustic panels, binderless composites

## Abstract

Effective sound absorption materials are essential for mitigating noise pollution in urban and industrial environments, which pose serious health risks to humans. This work develops a hierarchical natural fibre binderless composite based on porous luffa, modified with localised cellulose nanocrystals (CNCs), for application in acoustic panels. The impedance tube approach was employed to systematically evaluate sound absorption performance across a range of frequencies. Adding 3 wt.% and 7 wt.% CNCs to the porous luffa structure improved its sound absorption, especially in mid-to-high frequency areas. The binderless luffa panels with 3% CNC panels exhibited the most balanced performance across various thicknesses, while 7% CNC–luffa panels demonstrated excellent sound absorption averages across all frequency ranges, although increased rigidity and reflective tendencies were observed. The nano-modification successfully maintained the sound absorption coefficient with reduced panel thickness. This study establishes CNC-modified luffa composites as a sustainable and efficient alternative to conventional acoustic materials, leveraging renewable resources and lightweight characteristics. These findings highlight the potential of CNC-luffa composites for noise mitigation, paving the way for environmentally conscious acoustic solutions.

## 1. Introduction

Noise pollution from transport systems, industrial activities, and construction projects is a growing issue in urban and industrial areas. In England, nearly 40% of adults are exposed to road traffic noise levels that exceed 50 dB, which has been associated with significant health issues, such as sleep disturbances, cardiovascular diseases, and heightened stress levels [1]. Noise pollution can also cause non-auditory effects such as cognitive impairment and stress, contributing to various health complications [1,2]. Effective sound absorption is thus critical for creating environments that promote health and comfort, reducing the intensity of undesirable noise and improving safety in both residential and industrial environments.

Modern materials with enhanced sound absorption properties have become essential for addressing the health risks associated with noise pollution [3]. Flexible polyurethane (PU) foams with open cell structures are widely used in sectors such as the automotive industry due to their excellent sound absorption, achieved by converting sound waves into heat [4]. PU foams own their effective sound absorption to their porous structure, which dissipates sound energy through friction and viscous resistance. Several studies have focused on improving PU foams by incorporating fillers and nanofillers to enhance their sound absorption performance [5,6,7,8,9]. Factors such as catalyst types, foaming agents, and processing conditions also influence their acoustic properties [10,11,12].

While the porous structure of PU foams effectively scatters sound energy and can be tailored with nanofillers to enhance acoustic properties, several limitations persist. For example, pure PU foams often show inadequate sound absorption at low frequencies, while the use of synthetic components such as isocyanate poses significant environmental and health concerns [13].

In recent years, the demand for bio-based acoustic materials has grown rapidly due to their renewable nature, lower resource requirements, and reduced environmental footprint compared to synthetic options like polyurethane (PU) foam. Furthermore, natural fibres, often derived as agricultural byproducts, offer significant cost advantages over synthetic counterparts, making them a sustainable and economically viable alternative [14,15,16,17,18]. Nevertheless, research on porosity, density, and nanofillers in PU foam offers valuable insights for improving natural fibre acoustic panels. Natural fibre panels can enhance sound absorption by optimising interconnected voids between fibres, adjusting the size and distribution of pores to create longer routes for sound waves, increasing energy dissipation and improving overall sound absorption. These mechanisms closely resemble those observed in PU foams.

The porous and hierarchical structures of many natural materials made them an interesting option for sound absorption, while natural fibre such as coir, cork, kenaf, hemp, palm, tea, jute, ramie, and sisal offer promising alternatives, with sound absorption coefficients (SAC) and noise reduction capabilities extensively studied [19,20,21,22,23,24,25,26]. Key factors influencing SAC include air permeability, density, porosity, cell size, tortuosity, material thickness, and fibre orientation. Additionally, strategies such as single- or multilayer constructions, variations in sample preparation, and the incorporation of reinforcing materials have been explored to enhance the acoustic performance of natural fibre panels [24].

Several studies have examined how natural fibre treatments, binder types, and sample configurations influence sound absorption. Taban et al. reported that increasing the thickness and bulk density of kenaf fibre panels considerably enhanced SAC, especially at higher frequencies [27]. Sari et al. demonstrated that alkali treatment of corn husk fibres increased airflow resistivity and SAC values, highlighting the importance of treatment concentrations and airflow characteristics in optimising acoustic performance [28]. Putra et al. demonstrated that increasing the density of pineapple leaf fibres (PALF) increased SAC [29], while Santoni et al. and Xiang et al. emphasised the role of density and thickness, noting that higher density can either enhance or hinder sound absorption depending on the balance between porosity and fibre compaction [30,31]. Kassim et al. found that adding a thin kapok layer to coir fibres broadened the sound absorption bandwidth and enhanced performance at lower frequencies [32,33].

Luffa is a naturally porous and hollow fibrous material with a unique cellular structure, making it lightweight but mechanically weaker than other natural fibres such as jute or flax [16,34,35]. Despite its highly porous structure, which suggests excellent potential for sound absorption, limited research has explored the acoustic properties of luffa fibre composites. Halashi et al. reported that increasing the thickness and density of luffa panels enhances the SAC and sound absorption average (SAA), especially at lower frequencies [20]. Ayu et al. found that the multidirectional pores in luffa facilitated sound wave passage, resulting in reduced SAC at low frequencies but effective performance at mid frequencies, suggesting its potential for internal sound insulation [36]. Koruk and Genc demonstrated that pure luffa fibres have good mid-frequency absorption, with linen coverings further improving SAC across all frequencies. However, they noted that adding epoxy reduces SAC due to increased stiffness, demonstrating the effect of structural changes on acoustic characteristics [37].

Previous studies on luffa-based composites highlight several challenges for acoustic applications, particularly their inadequate sound absorption at low and mid frequencies. Achieving effective performance often requires thick panels (40–50 mm), which limits their suitability for many applications. Additionally, the lower mechanical strength of luffa fibres compared to other natural fibres restricts their use in structural contexts. While binders such as PVA and epoxy improve mechanical properties, they can reduce sound absorption, especially at lower frequencies. Luffa’s porous structure effectively scatters high-frequency sound waves, but struggles with low-frequency absorption due to the lower resistance offered to longer wavelengths. Optimising binder content remains another challenge, as excessive amounts compromise acoustic performance despite enhancing rigidity.

Nanofillers offer a promising solution to the limited low-frequency absorption capabilities of natural fibre panels. For instance, multiwall carbon nanotubes and graphene oxide enhance the acoustic properties of PU foams by increasing tortuosity, creating more uneven pathways for sound propagation and amplifying energy dissipation at lower frequencies [7,9,38,39]. Similarly, incorporating nanofillers into natural fibre panels can create a more intricate internal structure, extending the travel paths of sound waves and improving acoustic efficiency.

To date, no studies have explored the use of nanofillers to tailor the interface and tune the acoustic properties of luffa fibres. This gap is especially notable for green nanofillers like cellulose nanocrystals (CNC), which offer a sustainable alternative with a high surface area [40,41]. CNCs are expected to adhere to the cellular walls of luffa fibres, increasing the density and hierarchy of the internal network. These nanomodifications to the micro-architecture may alter sound wave interference and absorption, leading to a change in sound absorption coefficients.

This study develops hierarchical natural fibre composites based on luffa and CNC. CNC, a sustainable nanofiller with high affinity to natural fibres, were used to modify the surface of luffa fibres, increasing the surface area and roughness of luffa cavities to improve sound absorption. Panels with various CNC loadings (between 3 and 7 wt.%) and thicknesses (from 4 to 12 mm) were fabricated. The binding of luffa layers was achieved without additional binders by utilising the softening of lignin [42] within the luffa structure at hot press temperatures above 100 °C to enable interlocking, alongside the formation of hydrogen bonds between luffa fibres. Acoustic properties were characterised using an impedance tube method, systematically evaluating the effects of CNC on sound absorption efficiency of developed binderless natural fibre panels.

## 2. Experimental

### 2.1. Luffa and CNC Modification

Luffa sponges, sourced commercially and derived from the matured fruit of the luffa plant, served as the primary material. The sponges had an average length of 10 cm and a diameter of 6 cm. Cellulose nanocrystals (CNC) in powder form were purchased from CelluForce (Montreal, Canada, NCV100-NASD90). CNC solutions were prepared at concentrations between 1 wt.% and 3 wt.% in water. The CNC suspensions (500 mL total volume) were prepared using magnetic stirring for 2 h, followed by probe sonication at 3000 J/g energy with 20% amplitude (2 s on, 2 s off). Luffa sponges were pre-dried at 80 °C for 24 h to remove moistures before immersion in these solutions for one hour, followed by drying at room temperature for 24 h and subsequent oven-drying at 80 °C for an additional 24 h to ensure the complete removal of moisture.

### 2.2. Sample Preparation for Sound Absorption Test

**Figure 1:**
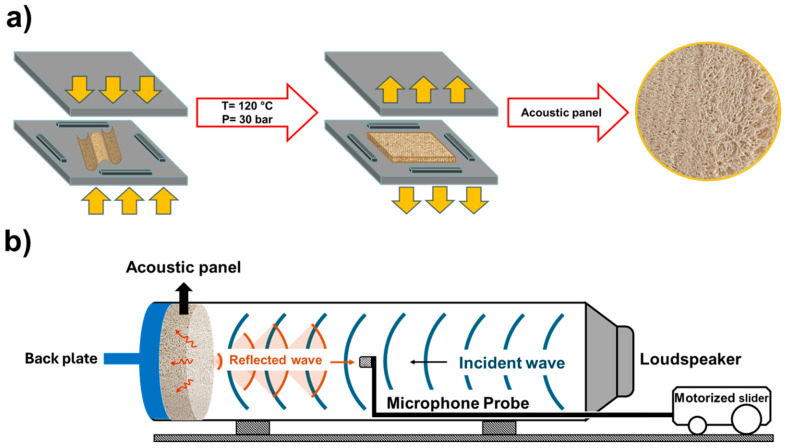
(**a**) Schematic illustration of the impedance tube used in this study. (**b**) Schematic of the fabrication process for panels using three halved luffas per layer, compression moulded. Applicable to neat and CNC-coated luffa (pre-treated by dip coating in CNCs solution).

Cylindrical luffa sponges were halved longitudinally, and residual seeds were removed. Each layer consists of three halved luffas placed next to each other, with desired sample thicknesses ranging from 4 mm to 12 mm achieved by layering different multiple sponges during compression moulding and with spacers used to control thickness. The desired number of luffa layers were compression-moulded into flat sheets using a Dr. Collin P300E press (Maitenbeth, Germany) at 120 °C and 30 bar pressure for 20 min. The resulting sheets were cut into 94 mm diameter discs for acoustic testing (Figure 1a).

### 2.3. Bulk Density and Weight Measurement

The density of samples with varying thicknesses and layers was calculated by dividing the mass of the disc (g) by its volume (cm^3^). Average density values were obtained from three replicates (density data are provided in Table S1 of the Supplementary Information). CNC loading was calculated based on the difference in dried sample mass before and after immersion in CNC solutions, using averaged values for four replicates. CNC loading on the dried modified luffa samples was approximately 3% for the 1% CNC solution and 7% for the 3% CNC solution, as shown in Table 1. The CNC loadings are referred to in the final composite panels in the subsequent sections.

### 2.4. Scanning Electron Microscopy

The morphology of the unmodified and CNC–luffa surfaces was examined using a Scanning Electron Microscope (SEM) (FEI Inspect-F, Eindhoven, The Netherlands) to examine the impact of the CNC coating on the surfaces and structures of the luffa samples.

### 2.5. Sound Absorption Measurements

Sound absorption tests were performed using the one-microphone impedance tube method in accordance with ASTM C384 [43] (Figure 1b). The setup consisted of a polycarbonate tube with a wall thickness of 3 mm, an inner diameter of 94 mm, and a length of 100 cm. Circular luffa samples (94 mm in diameter) were inserted into the tube and sealed externally with an 8 mm thick polycarbonate backing plate. Experiments were conducted at 20 °C and a relative humidity of 51%. A loudspeaker positioned at one end of the tube generated sinusoidal sound waves using a function generator (RS PRO GFG-8216A). The maximum (V_max_) and minimum (V_min_) voltage amplitudes were recorded as the microphone moved along the tube using an oscilloscope (RS PRO RSDS 1052 DL + 50MHz). The sound absorption coefficient (SAC) was calculated using standing wave ratio SWR(x) at position x and SAC (α_n_), as derived from Equations (1) and (2).

(1) $$SWR(x)=V_{max}(x)/V_{min}(x)$$

(2) $$\alpha_{n}=1-((SWR-1)/({SWR+1))}^{2}$$

The SAC (α_n_) was measured over a frequency range from 315 to 2000 Hz. The Sound Absorption Average (SAA) was calculated within this range, following ASTM C423 [44] with minor modifications to accommodate the lowest frequency tested and the resonance frequencies of the materials in this study. Additional details regarding these modifications are provided in the Supplementary Materials.

## 3. Results and Discussion

### 3.1. Morphologies of Neat and Modified Luffa

**Figure 2:**
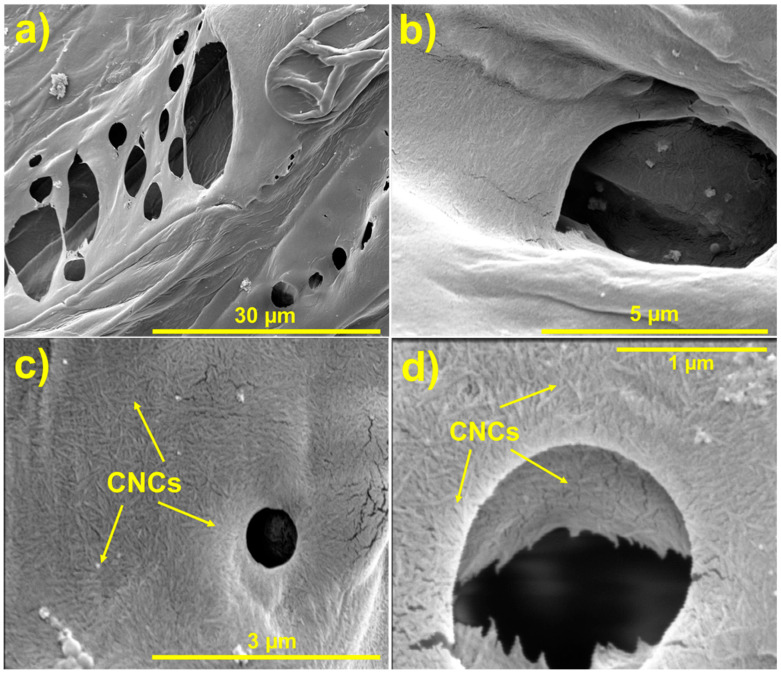
SEM images of neat luffa and 3 wt.% CNC-coated luffa. Images (**a**,**b**) show the highly porous structure of neat luffa with relatively large voids and smooth surfaces, while (**c**,**d**) show the 3 wt.% CNC modified luffa samples with rougher surfaces and reduced void dimensions.

Figure 2 shows the morphologies of both neat and CNC-modified luffa samples. The neat luffa (Figure 2a,b) exhibits a naturally porous structure with large irregular voids and a relatively smooth internal surface. The neat luffa’s relatively large voids provide limits resistance to sound propagation, which is a likely contributor to their sound absorption performance at low frequencies. In contrast, the CNC-modified luffa samples (Figure 2c,d) demonstrate substantial morphological changes, with CNC coating forming a denser and more hierarchical structure. The deposited CNCs adhere to the pore walls, creating rougher and more tortuous pathways. The modification increases the surface areas within the luffa samples, with certain cavities been bridged with CNCs exposed at cavities. There was no evidence of CNC removal during the sample preparation, confirming the effective bonding between CNC and luffa fibres due to the formation of hydrogen bonds. With such hierarchical microstructure observed, it is expected to have enhanced tortuosity and impedance of sound waves travelling through the materials.

### 3.2. Sound Absorption Properties

The Sound Absorption Coefficient (SAC) and Sound Absorption Average (SAA) were systematically analysed for unmodified and CNC-modified luffa samples, considering various thicknesses, layer configurations, and CNC concentrations. Low-frequency noise, typically in the range of 20–500 Hz, can cause significant physiological and psychological effects [45,46], while mid-frequency noise (500–2000 Hz) often dominates environmental soundscapes such as traffic or machinery noise [47,48]. Thus, materials capable of reducing noise across these frequencies are critical. This study evaluates the acoustic performance of luffa-based binderless panels with CNC modifications, aiming to optimise their performance through hierarchical structures.

**Effect of thickness on SAA**

**Figure 3:**
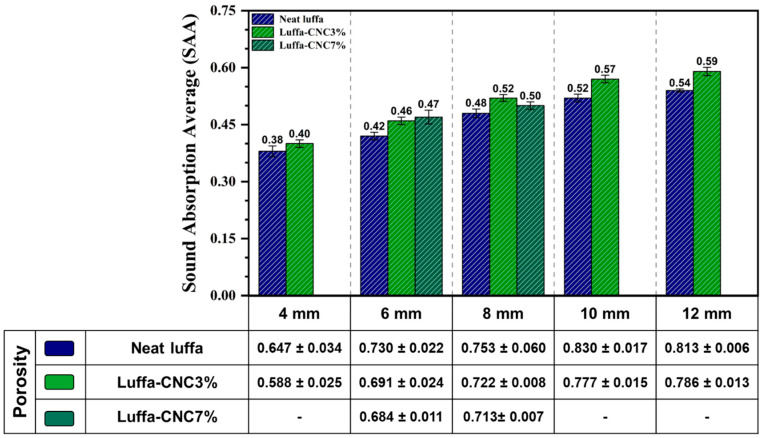
Sound Absorption Average (SAA) comparison for two layers of neat luffa and CNC3% and CNC7% luffa composites with different panel thicknesses, considering panel porosity.

Increasing sample thickness from 4 mm to 12 mm resulted in a clear increase in SAA values for both neat and CNC3% luffa samples (with a fixed two-layer configuration). For neat luffa, the SAA increased from 0.38 at 4 mm to 0.54 at 12 mm thickness, while CNC3% samples exhibited a corresponding increase from 0.40 to 0.59 (Figure 3). This trend reflects the increased capacity of thicker materials to attenuate low-frequency sounds, as longer sound wavelengths are effectively dissipated via friction and viscous losses within the porous structure. CNC-modified samples consistently outperformed neat samples, even at lower thicknesses, due to the enhanced interaction between sound waves and the CNC-enriched microstructures (Figure 2c,d). The deposited CNCs on luffa forms a rough, dense coating around the pore walls, creating a tortuous pathway for sound waves, enhancing absorption through vibrational damping.

It is important to note that while nanomaterials can enhance acoustic properties, the shape and structure of the nanofillers play a crucial role. It has been reported that nanomaterials with a layered structure, such as nanoclay and graphene nanoplatelets, are particularly effective in improving transmission loss [49]. In contrast, nanomaterials with a spherical structure, like nano silica, are more beneficial for sound absorption [49]. In this work, the CNCs surrounding the pores behaved similarly to a chord-like substance, capable of vibrating when impacted by sound energy, effectively damping sound waves and improving absorption. However, at higher CNC content (CNC7%), the material within some cells appears to form a more rigid surface, potentially reflecting sound waves, which could explain the observed decrease in sound absorption averages.

**Frequency-dependent sound absorption**

**Figure 4:**
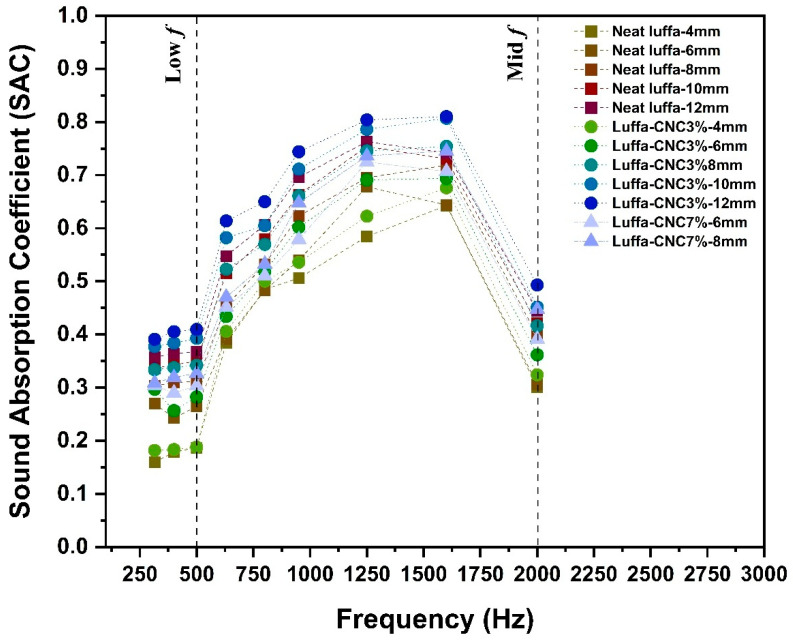
Sound Absorption Coefficient (SAC) of neat, CNC3%, and CNC7% luffa composites for the two-layer configuration, with thicknesses ranging from 4 mm to 12 mm for most cases.

Both neat and CNC-modified luffa samples demonstrated their highest SAC values at mid-frequency ranges (1250–1600 Hz), where sound energy is effectively dissipated due to the interaction between sound waves and the internal structures (Figure 4). Thicker panels, particularly those with a 12 mm thickness, demonstrated the highest SAC values across the frequency spectrum. This supports the principle that thicker materials dissipate sound more effectively by allowing for longer sound waves travel path, promoting energy loss through viscous and thermal interactions [27,50]. The 12 mm neat and 3%CNC luffa achieved average Noise Reduction Coefficient (NRC) of 0.54 and 0.59, respectively, outperforming thinner counterparts. However, the practicality and resource efficiency of thinner panels motivated further investigation into 8 mm configurations. It is also worth noting that predictive models can be used to deepen insights into the acoustic properties of CNC-modified luffa panels. For instance, the Delany–Bazley model offers a theoretical framework for comprehending the propagation and absorption of sound in porous media, covering the relationship between acoustic absorption and material parameters such as bulk density, porosity, and flow resistivity [51,52]. For CNC-modified luffa panels, decreased porosity is likely to increase flow resistivity, which in turn enhances sound absorption, particularly at mid-to-high frequencies. The Delany–Bazley model could be implemented in subsequent investigations to quantitatively characterise the acoustic properties of these materials.

Despite reduced thickness, 8 mm CNC3% panels achieved a SAC of 0.75 at both 1250 Hz and 1600 Hz, comparable to the thicker 12 mm panels, highlighting the effective enhancement on sound absorption by the introduction of CNC without increasing the overall thickness. Interestingly, CNC7% samples exhibited slightly lower SAC values than CNC3% due to reduced porosity, which could be attributed to the increased rigidity and potential sound reflection, particularly in the mid-frequency range.

The findings highlight the potential of 8 mm panels as a sustainable alternative for acoustic applications, balancing performance and material use. These panels achieved superior sound absorption efficiency due to the hierarchical internal structure enabled by CNC incorporation. The intermediate thickness of 8 mm panels allows for multi-layer configurations, providing flexibility for practical acoustic panel designs. This makes them an ideal candidate for exploring layering effects, which will be discussed in the subsequent section.

**Figure 5:**
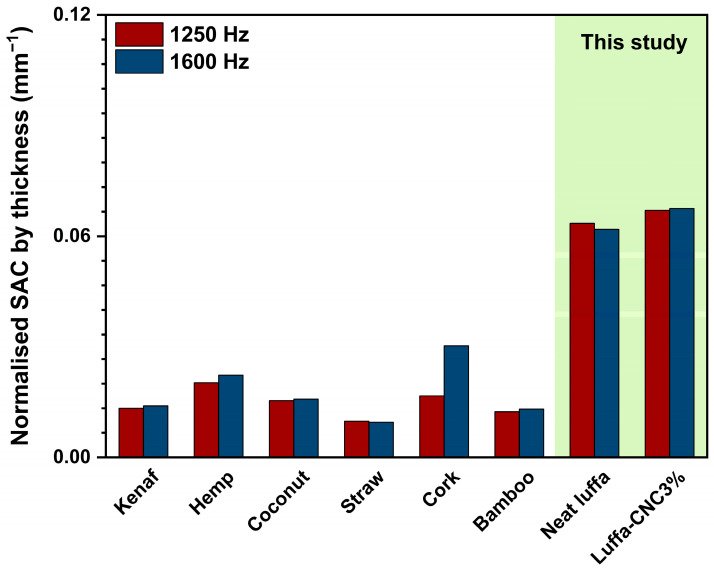
A comparison plot of reported normalised sound absorption coefficient (SAC) of 1250 Hz and 1600 Hz for various natural fibre composites from the literature [53,54], with the results from neat luffa and luffa CNC3% composites in this study for these two selected frequencies.

Figure 5 presents a comparative summary of normalised SAC for both the literature-reported values [53,54] and CNC3% luffa composites, demonstrating the effectiveness of CNC inclusion in enhancing sound absorption. The hierarchical structure and tortuosity introduced by CNC particles not only increased energy dissipation, but also allowed for the material to maintain competitive performance while retaining sustainability and lightweight characteristics.

**Effect of layering on SAC**

**Figure 6:**
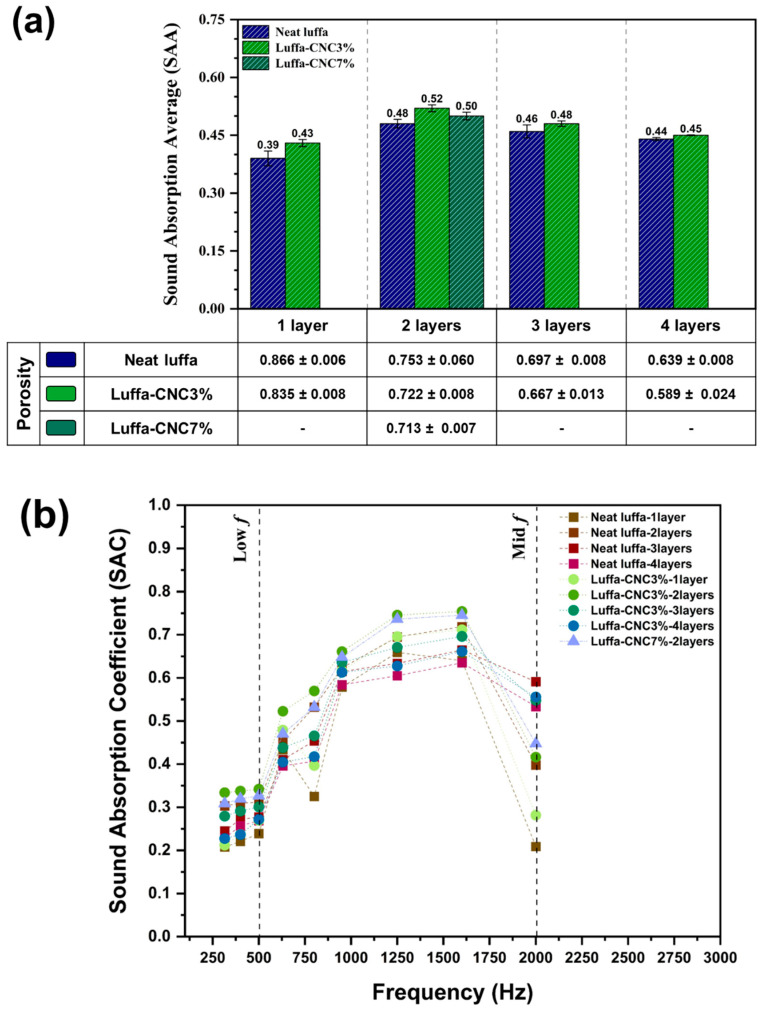
(**a**) Sound Absorption Average and porosity comparison for neat, CNC3%, and CNC7% luffa panels with various numbers of layers at 8 mm thickness. (**b**) Sound Absorption Coefficient comparison of neat luffa, CNC3%, and CNC7% luffa composites for 8 mm thickness panels with varying numbers of layers.

Clearly, layering influenced SAC and SAA values, with clear evidence of an optimal layer configuration for maximising acoustic performance. For 8 mm panels, the effect of layering was examined across configurations with from one to four layers. In neat luffa panels, SAA increased from 0.39 (1 layer) to 0.48 (2 layers) before decreasing to 0.44 for 4 layers, highlighting the importance of balancing porosity and density for maximum sound absorption (Figure 6a). A similar trend was observed in CNC 3% samples, where the SAA reached from 0.52 (two layers) from 0.43 (one layer), before declining with additional layers due to excessive compaction.

The frequency-dependent SAC values further confirmed these findings. At mid-frequency ranges between 1250 Hz and 1600 Hz, CNC3% panels with two-layer configurations achieved peak SAC values of 0.746 and 0.754 (Figure 6b), respectively. Additional layering (between three and four layers) reduced SAC due to decreased porosity and increased material rigidity, which limited the dissipation of sound energy. Specifically, Table S2 shows that porosity decreases from 0.866 (one layer) to 0.639 (four layers) for neat luffa, correlating with diminished SAC values. These observations highlight the importance of porosity–density balance in optimising acoustic properties, as excessive compaction hinders the effectiveness of sound wave attenuation.

To explore the effect of CNC content at the optimised 8 mm thickness and two-layer configuration, CNC3% was compared with CNC7% panels. As shown in Figure 6a,b, despite slightly reduced porosity (0.71 for CNC7% and 0.72 for CNC3%), CNC7% panels maintain high SAC values for CNC7% at 1250 Hz and 1600 Hz (0.736 and 0.745, respectively). These values were higher than those of neat luffa (0.70 and 0.72, respectively), but slightly lower than those of CNC3%, indicating that the increased CNC content might enhanced the material’s rigidity and reduced porosity to a degree that slightly limits mid-frequency sound absorption. These finding suggest that CNC modifications can enhance SAC and SAA through increased surface roughness and internal tortuosity, with optimal layering and CNC content playing important roles in acoustic properties. The correlation between sound absorption and porosity is well established, with a higher porosity generally enabling more effective dissipation of sound energy, particularly at low frequencies. This trend is confirmed for neat luffa samples, where quantitative porosity measurements (Table S2) indicate improved low-frequency sound absorption with increased porosity. However, in CNC-modified samples, the introduction of CNC reduces porosity and enhances internal tortuosity, leading to improved mid-to-high frequency sound absorption and increases density. The CNCs contribute to increased density and surface roughness, which scatter and interfere with sound waves more effectively at these frequencies. These findings highlight the dual role of CNCs in modifying structural properties to optimise acoustic performance. While increased CNC content enhances the rigidity and density of the material, this effect should be balanced with porosity to achieve the desired acoustic properties. Thus, the CNC3% specimen appears to provide a more favourable balance for broad-spectrum sound absorption, while CNC7% specimen offers advantages for targeted mid-to-high frequency performance. The interplay between CNC content, porosity, and density highlights the importance of carefully tailoring material parameters to meet specific acoustic application requirements.

## 4. Conclusions

This study demonstrates the potential of luffa sponge as an effective porous material for sound absorption applications, with significant performance enhancement achieved through cellulose nanocrystal (CNC) modifications. Among the tested configurations, luffa panels with 3 wt.% CNCs exhibited the most balanced and outstanding sound absorption capabilities, particularly at mid-frequency ranges (1250 Hz–1600 Hz). In contrast, CNC7% luffa panels showed enhanced sound absorption at higher frequencies, although the increased stiffness and reduced porosity associated with high CNC contents slightly limited performance at mid-frequency range. These findings highlight the importance of balancing porosity and material rigidity to optimise acoustic properties.

The results indicate that CNC modifications improve sound absorption even at relatively low panel thicknesses due to the increased surface roughness and tortuosity within the luffa structure. Increasing thickness generally improved SAC, but CNC modifications allowed for thinner panels to achieve comparable performance, demonstrating the value of CNC reinforcement for material efficiency and practicality.

CNC reinforcement not only enhances the acoustic performance of luffa-based materials, but also offers a sustainable and lightweight alternative for noise mitigation applications. The optimised CNC3% luffa composite provides a practical solution for sound absorption across a wide frequency range, balancing efficiency and performance, and outperforms many natural fibre-based materials reported in the literature. These findings establish CNC-modified luffa composites as a promising candidate for environmentally friendly acoustic insulation in industrial and residential settings.

## Figures and Tables

**Table 1:** Average mass before and after immersion of luffa samples, with CNC loading in panels calculated for 1% and 3% CNC solutions.

CNC Solution Concentration (wt.%)	Average Mass Before Immersion (g)	Average Mass After Immersion (g)	Average CNC Absorbed (g)	CNC Loading in Panels (wt.%)
1	5.65 ± 0.79	5.83 ± 0.79	0.18 ± 0.04	3.13 ± 0.61
3	5.41 ± 0.74	5.82 ± 0.72	0.41 ± 0.03	7.01 ± 1.10

## Data Availability

The raw data supporting the conclusions of this article will be made available by the authors on request.

## Supplementary Materials

The following supporting information can be downloaded at: https://www.mdpi.com/article/10.3390/polym17030281/s1, Figure S1: SAC curve for neat and CNCs modified luffa samples with two luffa layers across different thicknesses. Figure S2: SAC curve for neat and CNCs modified luffa samples with 8mm panel thickness from 1 layer luffa to four layers luffa thicknesses. Figure S3: SAC comparison for tests at 900 Hz, 950 Hz, 1000 Hz, 1050 Hz, and 1100 Hz. Table S1: The bulk density of various thickness and layer samples. Table S2: The porosity of various thickness and layer samples. Table S3: Sound absorption coefficient (α) for frequencies ranging from 315–2000 Hz for neat luffa, luffa CNC3%, and luffa CNC7% samples with thicknesses varying from 4 mm to 12 mm. Table S4: Sound Absorption Coefficient (SAC) for unmodified luffa, CNC3% and CNC7% luffa composites for 8mm thickness panels with varying layers from one to four luffa layers. References [34,55,56,57,58,59] are cited in the Supplementary Materials.
