# Supplementary material for: Hierarchical Natural Fibre Composites Based on Cellulose Nanocrystal-Modified Luffa Structures for Binderless Acoustic Panels
Source: Polymers (Basel). 2025 Jan 22;17(3):281. doi: 10.3390/polym17030281 (PMC11821052; doi:10.3390/polym17030281)
Supplement: Supplementary file 1 [file polymers-17-00281-s001.zip › polymers-3409679-supplementary.pdf]

Hierarchical natural fibre composites based on cellulose nanocrystals modified luffa structures for binderless acoustic panels

Shahed Ekbatani[1], Phattharasaya Rattanawongkun[2], Supattra Klayya[2], Dimitrios G. Papageorgiou[1], Nattakan Soykeabkaew[2], Han Zhang[1*]

[1] School of Engineering and Materials Science, Queen Mary University of London, London E1 4NS , United Kingdom

[2] School of Science, Mae Fah Luang University, 333 M1, Muang, Chiang Rai, 57100, Thailand

Supplementary Information

Table S1. The bulk density of various thickness and layer samples.

Sample	Sample weight (g)			Sample volume (cm³)			Sample density (g/cm³)
Neat luffa-2layers-4mm	9.010	10.430	9.100	30.884	30.155	30.597	0.312±0.030
Neat luffa-2layers-6mm	10.460	10.790	11.790	45.543	47.824	45.187	0.239±0.019
Neat luffa-2layers-8mm	11.990	11.860	15.750	60.450	66.666	56.719	0.218±0.053
Neat luffa-2layers-10mm	10.100	12.110	12.810	74.536	81.009	77.343	0.150±0.015
Neat luffa-2layers-12mm	13.250	15.320	14.890	83.341	91.273	88.412	0.165±0.005
Luffa CNC3%-2layers-4mm	10.920	10.750	11.880	30.762	29.739	29.895	0.371±0.023
Luffa CNC3%-2layers-6mm	11.950	12.490	12.890	45.476	46.157	42.407	0.279±0.022
Luffa CNC3%-2layers-8mm	13.470	14.290	13.140	53.919	55.260	53.886	0.251±0.007
Luffa CNC3%-2layers-10mm	14.020	14.440	14.840	74.940	71.276	69.344	0.201±0.014
Luffa CNC3%-2layers-12mm	14.970	15.320	16.890	80.935	81.882	82.117	0.193±0.011

Luffa CNC7%-2layers-6mm	12.930	13.640	13.040	45.094	44.901	45.412	0.293±0.010
Luffa CNC7%-2layers-8mm	14.940	15.267	15.650	57.359	57.918	57.370	0.266±0.006
Neat luffa-1layer-8mm	6.850	7.082	6.470	58.446	56.877	57.051	0.118±0.006
Neat luffa-3layers-8mm	15.450	15.830	16.030	59.395	58.848	58.381	0.268±0.007
Neat luffa-4layers-8mm	18.390	19.210	21.800	59.121	59.202	67.729	0.319±0.007
Luffa CNC3%-1layer-8mm	8.160	8.620	8.940	57.340	58.380	57.259	0.149±0.007
Luffa CNC3%-3layers-8mm	17.360	17.940	19.340	57.702	62.184	62.080	0.300±0.012
Luffa CNC3%-4layers-8mm	21.460	23.310	26.360	59.298	65.657	66.588	0.371±0.022

A noteworthy observation made on the SAC curves of the unmodified and CNC-modified samples was the presence of a drop at 1000 Hz (Figure S1 and Figure S2). The resonance effect, in which the frequency of the incoming sound corresponds with the natural resonance frequency of the material, is responsible for this decrease in SAC [1-4]. There is a drop in SAC at this frequency because sound energy is reflected rather than dissipated. The unmodified luffa samples showed a more noticeable decrease, but the CNC-modified samples showed a less noticeable drop. This suggests that the CNC reinforcement enhances the material's structural consistency and reduces the negative impact of resonance, thereby improving performance even at problematic frequencies like 1000 Hz. To confirm that 1000 Hz is the resonance frequency causing a drop in SAC, additional tests were conducted at 900 Hz, 950 Hz, 1050 Hz, and 1100 Hz, which did not exhibit similar drops, thereby proving the resonance effect.

To confirm that 1000 Hz is indeed the resonance frequency causing the drop in SAC, additional tests were conducted at 900 Hz, 950 Hz, 1050 Hz, and 1100 Hz. These tests did not exhibit similar drops in SAC, confirming that 1000 Hz is the resonance frequency. Based on these findings, it was decided to replace 1000 Hz with 950 Hz when calculating the sound absorption average (SAA) to avoid miscalculation due to the resonance-related drop at 1000 Hz.

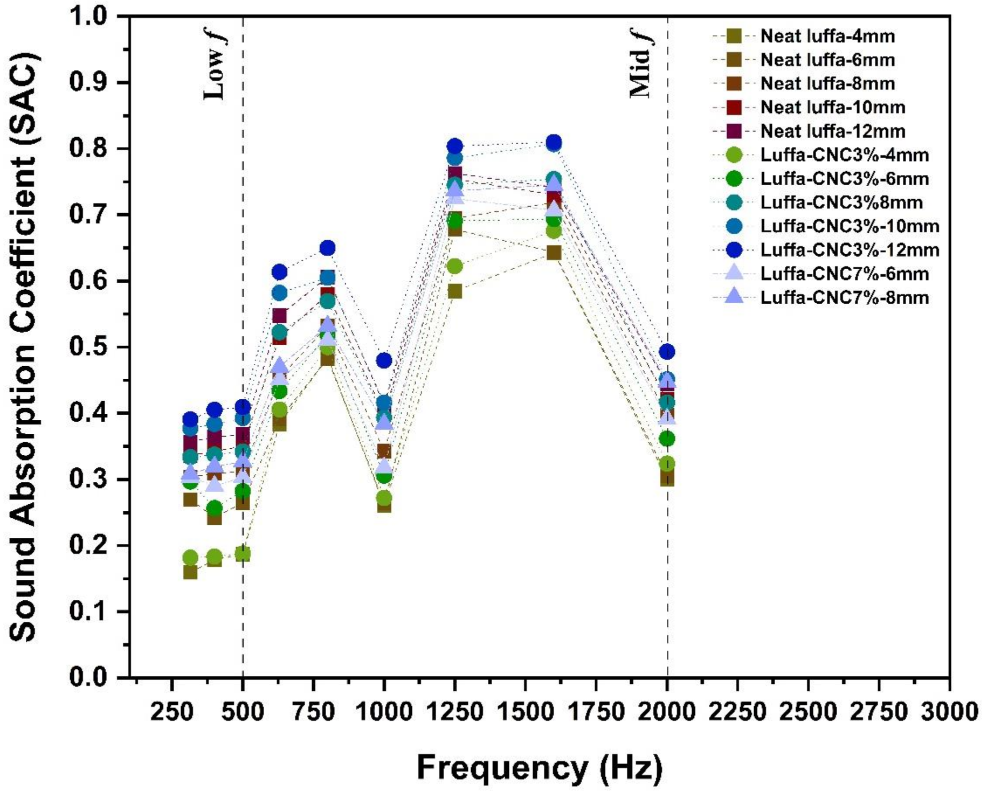

Figure S1: SAC curve for neat and CNCs modified luffa samples with two luffa layers across different thicknesses showing a drop at 1000 Hz, indicating a resonance frequency.

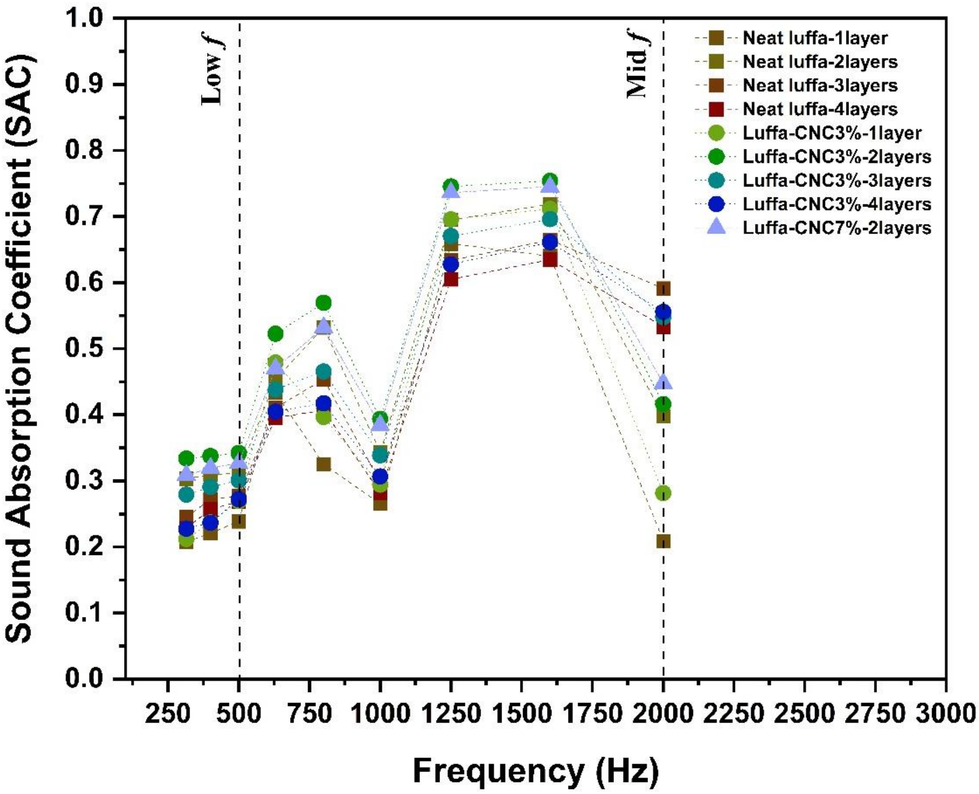

Figure S2: SAC curve for neat and CNCs modified luffa samples with 8mm panel thickness from 1 layer luffa to four layers luffa thicknesses, showing a drop at 1000 Hz, indicating a resonance frequency.

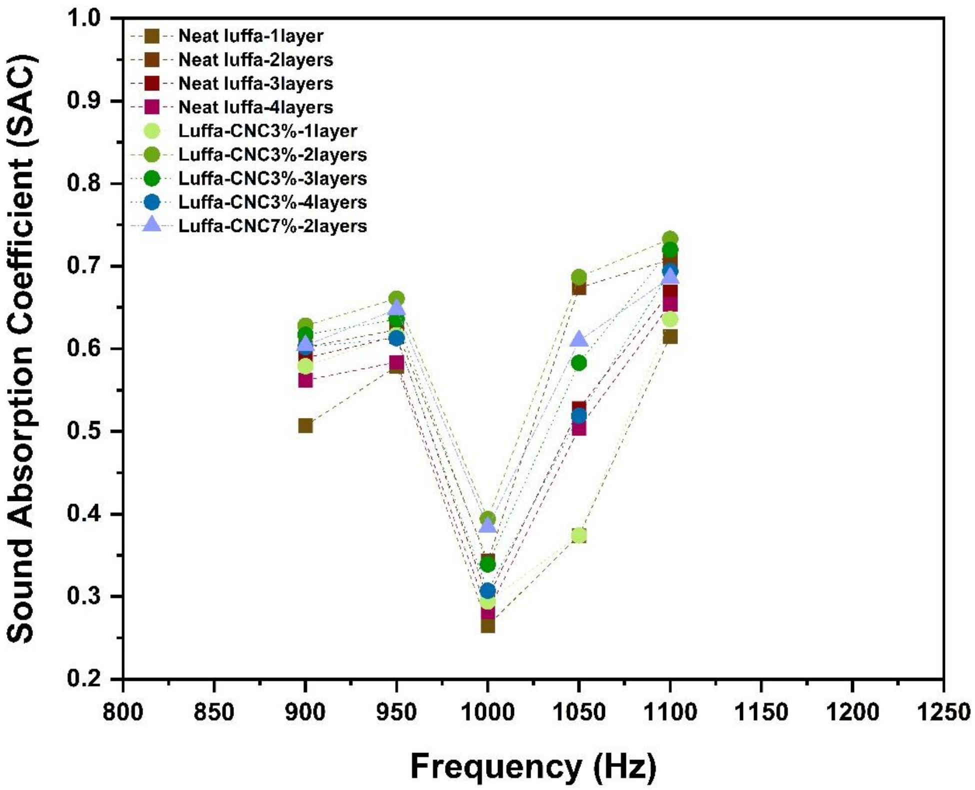

Figure S3: SAC comparison for tests at 900 Hz, 950 Hz, 1000 Hz, 1050 Hz, and 1100 Hz. The results confirm that the drop at 1000 Hz is caused by resonance, as no significant drop is observed at the adjacent frequencies.

The porosity (ε) of the samples is calculated by dividing the void volume by the total volume of the material [5]. The relationship between bulk density (ρ_{bulk}) and fibre density (ρ_{fibre}) allows to calculate porosity. The formula for showing this relationship is:

$$\rho_{bulk} = (1 - \varepsilon)\rho_{fibre} \tag{S1}$$

This equation estimates porosity by comparing the sample's bulk density to the known density of luffa fibre (0.883 g/cm³) [6]. The difference between these densities represents the sample's interior voids, which have a direct impact on the material's acoustic properties.

Table S2. The porosity of various thickness and layer samples.

Sample	Sample density (g/cm³)	Density of fibre (g/cm³)	Porosity (ε)
Neat luffa-2layers-4mm	0.312±0.030	0.883	0.647 ± 0.034
Neat luffa-2layers-6mm	0.239±0.019	0.883	0.730 ± 0.022
Neat luffa-2layers-8mm	0.218±0.053	0.883	0.753 ± 0.060
Neat luffa-2layers-10mm	0.150±0.015	0.883	0.830 ± 0.017
Neat luffa-2layers-12mm	0.165±0.005	0.883	0.813 ± 0.006
Luffa CNC3%-2layers-4mm	0.371±0.023	0.902	0.588 ± 0.025
Luffa CNC3%-2layers-6mm	0.279±0.022	0.902	0.691 ± 0.024
Luffa CNC3%-2layers-8mm	0.251±0.007	0.902	0.722 ± 0.008
Luffa CNC3%-2layers-10mm	0.201±0.014	0.902	0.777 ± 0.015
Luffa CNC3%-2layers-12mm	0.193±0.011	0.902	0.786 ± 0.013
Luffa CNC7%-2layers-6mm	0.293±0.010	0.927	0.684 ± 0.011
Luffa CNC7%-2layers-8mm	0.266±0.006	0.927	0.713 ± 0.007
Neat luffa-1layer-8mm	0.118±0.006	0.883	0.866 ± 0.006
Neat luffa-3layers-8mm	0.268±0.007	0.883	0.697 ± 0.008
Neat luffa-4layers-8mm	0.319±0.007	0.883	0.639 ± 0.008
Luffa CNC3%-1layer-8mm	0.149±0.007	0.902	0.835 ± 0.008
Luffa CNC3%-3layers-8mm	0.300±0.012	0.902	0.667 ± 0.013
Luffa CNC3%-4layers-8mm	0.371±0.022	0.902	0.589 ± 0.024

Table S3. Sound absorption coefficient (α) for frequencies ranging from 315–2000 Hz for neat luffa, luffa CNC3%, and luffa CNC7% samples with thicknesses varying from 4 mm to 12 mm**.**

**Sample**	**Number of samples**	**Thickness (mm)**	**Number of layers**	**Frequency (Hz)**									**Sound Absorption Average (SAA)**
				**315**	**400**	**500**	**630**	**800**	**950**	**1250**	**1600**	**2000**	
				**Sound Absorption Coefficient (α)**									
**Neat luffa**	1	4	2	0.159	0.175	0.181	0.380	0.469	0.497	0.575	0.633	0.284	0.373
	2	4	2	0.165	0.192	0.199	0.405	0.505	0.513	0.614	0.669	0.310	0.397
	3	4	2	0.155	0.168	0.180	0.367	0.483	0.507	0.565	0.627	0.308	0.373
	Average			0.160± 0.005	0.179± 0.012	0.187± 0.011	0.384± 0.019	0.485± 0.018	0.506± 0.008	0.585± 0.026	0.643± 0.023	0.301± 0.015	**0.38± 0.014**
	1	6	2	0.272	0.246	0.274	0.405	0.497	0.568	0.678	0.669	0.310	0.436
	2	6	2	0.266	0.240	0.268	0.374	0.483	0.521	0.691	0.653	0.293	0.421
	3	6	2	0.270	0.241	0.252	0.395	0.469	0.532	0.665	0.609	0.315	0.416
	Average			0.270± 0.003	0.243± 0.003	0.265± 0.012	0.391± 0.016	0.483± 0.014	0.540± 0.025	0.678± 0.013	0.644± 0.031	0.306± 0.012	**0.424± 0.010**
	1	8	2	0.295	0.294	0.308	0.432	0.535	0.640	0.710	0.701	0.414	0.481
	2	8	2	0.301	0.314	0.314	0.432	0.523	0.628	0.683	0.691	0.372	0.473
	3	8	2	0.313	0.321	0.312	0.496	0.540	0.601	0.691	0.764	0.407	0.494
	Average			0.303± 0.009	0.309± 0.014	0.312± 0.003	0.454± 0.037	0.532± 0.009	0.623± 0.020	0.695± 0.014	0.719± 0.039	0.398± 0.022	**0.483± 0.011**
	1	10	2	0.337	0.348	0.337	0.501	0.556	0.640	0.723	0.739	0.413	0.510
	2	10	2	0.339	0.347	0.348	0.521	0.584	0.667	0.764	0.728	0.420	0.524
	3	10	2	0.332	0.332	0.365	0.524	0.599	0.682	0.776	0.723	0.431	0.529
	Average			0.336± 0.004	0.343± 0.009	0.350± 0.014	0.515± 0.013	0.579± 0.022	0.663± 0.021	0.754± 0.028	0.730± 0.008	0.421± 0.009	**0.521± 0.010**
	1	12	2	0.354	0.367	0.370	0.532	0.607	0.658	0.776	0.743	0.441	0.539
	2	12	2	0.360	0.365	0.362	0.549	0.602	0.730	0.763	0.732	0.462	0.547
	3	12	2	0.357	0.360	0.371	0.562	0.609	0.701	0.750	0.750	0.430	0.543
	Average			0.357± 0.003	0.364± 0.003	0.368± 0.005	0.548± 0.015	0.606± 0.004	0.696± 0.036	0.763± 0.013	0.742± 0.009	0.444± 0.017	**0.543± 0.004**
**Luffa-CNC3%**	1	4	2	0.181	0.184	0.193	0.402	0.462	0.538	0.614	0.627	0.331	0.393
	2	4	2	0.181	0.182	0.174	0.410	0.549	0.532	0.589	0.681	0.306	0.400
	3	4	2	0.183	0.183	0.196	0.405	0.490	0.538	0.665	0.720	0.336	0.413
	Average			0.182± 0.001	0.183± 0.001	0.188± 0.012	0.406± 0.004	0.500± 0.044	0.536± 0.003	0.622± 0.039	0.676± 0.046	0.324± 0.016	**0.402± 0.010**
	1	6	2	0.294	0.267	0.282	0.426	0.532	0.619	0.710	0.678	0.373	0.465
	2	6	2	0.298	0.249	0.279	0.426	0.476	0.595	0.640	0.712	0.354	0.448
	3	6	2	0.298	0.254	0.286	0.450	0.547	0.590	0.723	0.691	0.358	0.466
	Average			0.297± 0.002	0.257± 0.009	0.282± 0.003	0.434± 0.014	0.518± 0.038	0.602± 0.016	0.691± 0.045	0.694± 0.017	0.362± 0.010	**0.460± 0.010**
	1	8	2	0.337	0.333	0.325	0.512	0.556	0.652	0.736	0.735	0.413	0.511
	2	8	2	0.343	0.342	0.345	0.508	0.587	0.665	0.738	0.793	0.438	0.529
	3	8	2	0.321	0.338	0.356	0.548	0.566	0.665	0.763	0.735	0.398	0.521
	Average			0.334± 0.011	0.338± 0.005	0.342± 0.016	0.523± 0.022	0.570± 0.016	0.661± 0.007	0.746± 0.015	0.754± 0.034	0.416± 0.020	**0.520± 0.009**
	1	10	2	0.381	0.387	0.391	0.607	0.609	0.710	0.810	0.802	0.454	0.572
	2	10	2	0.360	0.377	0.384	0.578	0.591	0.694	0.785	0.802	0.421	0.555
	3	10	2	0.391	0.387	0.403	0.561	0.615	0.730	0.763	0.816	0.479	0.572
	Average			0.377± 0.016	0.384± 0.006	0.393± 0.010	0.582± 0.023	0.605± 0.012	0.711± 0.018	0.786± 0.024	0.807± 0.008	0.451± 0.029	**0.566± 0.010**
	1	12	2	0.374	0.399	0.391	0.607	0.640	0.740	0.785	0.798	0.473	0.579
	2	12	2	0.401	0.412	0.419	0.601	0.655	0.731	0.816	0.816	0.493	0.594
	3	12	2	0.397	0.404	0.419	0.633	0.655	0.760	0.810	0.816	0.514	0.601

	Average			0.391± 0.015	0.405± 0.007	0.409± 0.016	0.614± 0.017	0.650± 0.009	0.744± 0.015	0.804± 0.016	0.810± 0.011	0.493± 0.020	**0.591± 0.011**
**Luffa-CNC7%**	1	6	2	0.319	0.270	0.335	0.512	0.540	0.595	0.701	0.720	0.438	0.492
	2	6	2	0.301	0.297	0.300	0.443	0.476	0.565	0.750	0.736	0.377	0.472
	3	6	2	0.291	0.304	0.275	0.400	0.517	0.578	0.723	0.665	0.360	0.457
	Average			0.304± 0.014	0.290± 0.018	0.303± 0.030	0.452± 0.057	0.511± 0.032	0.579± 0.015	0.725± 0.024	0.707± 0.037	0.391± 0.041	**0.474± 0.018**
	1	8	2	0.284	0.308	0.332	0.473	0.534	0.640	0.764	0.757	0.458	0.506
	2	8	2	0.297	0.312	0.319	0.443	0.515	0.665	0.688	0.750	0.448	0.493
	3	8	2	0.345	0.337	0.331	0.495	0.547	0.640	0.757	0.728	0.438	0.513
	Average			0.309± 0.032	0.319± 0.016	0.327± 0.007	0.470± 0.026	0.532± 0.016	0.648± 0.014	0.736± 0.042	0.745± 0.015	0.448± 0.010	**0.504± 0.010**

Table S4. Sound Absorption Coefficient (SAC) for unmodified luffa, CNC3% and CNC7% luffa composites for 8mm thickness panels with varying layers from one to four luffa layers.

**Sample**	**Number of samples**	**Thickness (mm)**	**Number of layers**	**Frequency (Hz)**									**Sound Absorption Average (SAA)**
				**315**	**400**	**500**	**630**	**800**	**950**	**1250**	**1600**	**2000**	
				**Sound Absorption Coefficient (α)**									
**Neat luffa**	1	8	1	0.215	0.236	0.253	0.476	0.325	0.561	0.699	0.699	0.224	0.410
	2	8	1	0.204	0.207	0.231	0.406	0.292	0.585	0.609	0.640	0.204	0.375
	3	8	1	0.205	0.220	0.233	0.418	0.282	0.589	0.669	0.581	0.198	0.377
	Average			0.20± 0.006	0.22± 0.015	0.23± 0.012	0.43± 0.037	0.32± 0.023	0.57± 0.015	0.65± 0.046	0.64± 0.059	0.209± 0.013	**0.390± 0.019**
	1	8	2	0.295	0.294	0.308	0.432	0.535	0.640	0.710	0.701	0.414	0.481
	2	8	2	0.301	0.314	0.314	0.432	0.523	0.628	0.683	0.691	0.372	0.473
	3	8	2	0.313	0.321	0.312	0.496	0.540	0.601	0.691	0.764	0.407	0.494
	Average			0.30± 0.009	0.30± 0.014	0.31± 0.003	0.45± 0.037	0.53± 0.009	0.62± 0.020	0.69± 0.014	0.71± 0.039	0.398± 0.022	**0.483± 0.011**
	1	8	3	0.248	0.259	0.267	0.401	0.415	0.628	0.626	0.627	0.575	0.449
	2	8	3	0.233	0.276	0.276	0.412	0.395	0.578	0.614	0.669	0.585	0.449
	3	8	3	0.254	0.282	0.289	0.418	0.454	0.640	0.661	0.699	0.614	0.479
	Average			0.24± 0.011	0.27± 0.012	0.27± 0.011	0.41± 0.009	0.45± 0.030	0.61± 0.033	0.63± 0.025	0.66± 0.037	0.591± 0.020	**0.463± 0.017**
	1	8	4	0.238	0.267	0.269	0.401	0.430	0.545	0.588	0.656	0.543	0.437
	2	8	4	0.218	0.247	0.267	0.395	0.388	0.566	0.624	0.624	0.536	0.430
	3	8	4	0.229	0.256	0.271	0.392	0.402	0.640	0.602	0.624	0.521	0.437
	Average			0.22± 0.010	0.25± 0.010	0.26± 0.002	0.39± 0.004	0.40± 0.021	0.58± 0.050	0.60± 0.018	0.63± 0.018	0.533± 0.012	**0.435± 0.004**
**Luffa-CNC3%**	1	8	1	0.216	0.240	0.272	0.505	0.395	0.628	0.698	0.716	0.284	0.439
	2	8	1	0.215	0.243	0.280	0.483	0.418	0.617	0.705	0.720	0.288	0.441
	3	8	1	0.209	0.231	0.258	0.476	0.377	0.606	0.685	0.699	0.272	0.424
	Average			0.21± 0.004	0.23± 0.009	0.26± 0.016	0.47± 0.005	0.39± 0.021	0.61± 0.011	0.69± 0.010	0.71± 0.011	0.282± 0.008	**0.433± 0.009**
	1	8	2	0.337	0.333	0.325	0.512	0.556	0.652	0.736	0.735	0.413	0.511
	2	8	2	0.343	0.342	0.345	0.508	0.587	0.665	0.738	0.793	0.438	0.529
	3	8	2	0.321	0.338	0.356	0.548	0.566	0.665	0.763	0.735	0.398	0.521
	Average			0.33± 0.011	0.33± 0.005	0.34± 0.016	0.52± 0.022	0.57± 0.016	0.66± 0.007	0.74± 0.015	0.75± 0.034	0.416± 0.020	**0.520± 0.009**

	1	8	3	0.293	0.290	0.298	0.438	0.456	0.640	0.652	0.640	0.549	0.473
	2	8	3	0.284	0.297	0.307	0.450	0.482	0.617	0.698	0.716	0.532	0.487
	3	8	3	0.261	0.286	0.297	0.428	0.459	0.652	0.662	0.732	0.562	0.482
	Average			0.27± 0.016	0.29± 0.005	0.30± 0.006	0.43± 0.011	0.46± 0.014	0.63± 0.018	0.67± 0.024	0.69± 0.049	0.548± 0.015	**0.481± 0.007**
	1	8	4	0.232	0.244	0.260	0.406	0.426	0.617	0.619	0.654	0.546	0.445
	2	8	4	0.227	0.227	0.278	0.395	0.431	0.606	0.624	0.669	0.556	0.446
	3	8	4	0.224	0.238	0.278	0.412	0.395	0.617	0.640	0.661	0.565	0.448
	Average			0.22± 0.004	0.23± 0.008	0.27± 0.011	0.40± 0.009	0.41± 0.020	0.61± 0.006	0.62± 0.011	0.66± 0.007	0.556± 0.009	**0.446± 0.001**
**Luffa-CNC7%**	1	8	2	0.284	0.308	0.332	0.473	0.534	0.640	0.764	0.757	0.458	0.506
	2	8	2	0.297	0.312	0.319	0.443	0.515	0.665	0.688	0.750	0.448	0.493
	3	8	2	0.345	0.337	0.331	0.495	0.547	0.640	0.757	0.728	0.438	0.513
	Average			0.30± 0.032	0.31± 0.016	0.32± 0.007	0.47± 0.026	0.53± 0.016	0.64± 0.014	0.73± 0.042	0.74± 0.015	0.448± 0.010	**0.504± 0.010**

References

[1] Hasani Baferani A, Ohadi AR, Keshavarz R. Toward mechanistic understanding of the relationship between the sound absorption and the natural and resonant frequencies of porous media. J Acoust Soc Am. 2016;140(6):4246.

[2] Otaru AJ. Review on the Acoustical Properties and Characterisation Methods of Sound Absorbing Porous Structures: A Focus on Microcellular Structures Made by a Replication Casting Method. Metals and Materials International. 2019;26(7):915-32.

[3] Bravo T, Maury C, Pinhède C. Vibroacoustic properties of thin micro-perforated panel absorbers. The Journal of the Acoustical Society of America. 2012;132(2):789-98.

[4] FROMMHOLD W, FUCHS HV, SHENGt S. ACOUSTIC PERFORMANCE OF MEMBRANE ABSORBERS. Journal of Sound and Vibration 1994;170(S):621-36.

[5] Abdi DD, Monazzam M, Taban E, Putra A, Golbabaei F, Khadem M. Sound absorption performance of natural fiber composite from chrome shave and coffee silver skin. Applied Acoustics. 2021;182.

[6] Adeyanju CA, Ogunniyi S, Ighalo JO, Adeniyi AG, Abdulkareem SA. A review on Luffa fibres and their polymer composites. Journal of Materials Science. 2020;56(4):2797-813.
